# Penetrating cardiac trauma: analysis of 240 cases from a hospital in Bogota, Colombia

**DOI:** 10.1186/s13017-017-0138-1

**Published:** 2017-06-12

**Authors:** Andres Isaza-Restrepo, Dínimo José Bolívar-Sáenz, Marcos Tarazona-Lara, José Rafael Tovar

**Affiliations:** 1 Escuela de Medicina y Ciencias de la Salud, Universidad del Rosario, Carrera 24 No 63C - 69 Barrio Siete de Agosto, Bogotá, DC Colombia; 2 Méderi Hospital Universitario Mayor, Carrera 24 No 63C - 69 Barrio Siete de Agosto, Bogotá, DC Colombia; 3 Hospital Occidente de Kennedy, Bogotá, D.C. Colombia; 4 Escuela de Estadística, Facultad de Ingenierías, Universidad del Valle, Santiago de Cali, Colombia

**Keywords:** Cardiac trauma, Penetrating chest wounds, Heart injury, Penetrating wounds, Cardiac tamponade, Pericardial window, Sternotomy, Case series

## Abstract

**Background:**

Trauma characteristics and its management is influenced by socioeconomic context. Cardiac trauma constitutes a challenge for surgeons, and outcomes depend on multiple factors including initial care, characteristics of the wounds, and surgical management.

**Methods:**

This is a retrospective cross-sectional case series of patients with penetrating cardiac injuries (PCI) from January 1999 to October 2009 who underwent surgery in a trauma referral center in Bogotá, Colombia. Demographic variables, trauma characteristics, treatment, and outcomes were analyzed.

**Results:**

The study included 240 cases: 96.2% males, mean age of 27.8 years. Overall mortality was 14.6%: 11.7% from stab wounds and 41.2% from gunshot wounds. Upon admission, 44% had a normal hemodynamic status and 67% had cardiac tamponade. About 32% had Grade II injuries and 29% Grade IV injuries. In 85% of the cases, there were ventricular compromise and 55% of patients had associated lesions. In 150 cases, a pericardial window was performed. Highest mortality occurred in wounds to the right atrium. In tamponade patients, mortality was 20% being higher for gunshot wounds (54.5%) than for stab wounds (18%) (*p* = 0.0120).

**Conclusions:**

The study evidenced predominance of stab wounds. Based on characteristics of the trauma, patients, and survival rate, there is most likely a high pre-hospitalization mortality rate. The difference in mortality due to stab wounds and those produced by gunshots was more related to technical difficulties of the surgical repair than with the type of injury established by the Injury Grading Scale. Mortality was higher in patients with cardiac tamponade. Surgical management was satisfactory using pericardial window as the diagnostic method and sternotomy as the surgical approach.

## Background

Due to high mortality rates, cardiac trauma management is a challenge for trauma teams. Based on the National Trauma Data Bank of the American College of Surgeons (ACS), Asensio et al. [1] calculated a 0.16% incidence of penetrating cardiac injury (PCI) admissions to trauma centers. Mandal and Sanusi [2] found that PCI occurred in 6.4% of the penetrating chest injuries, one of the most frequently injured body segments.

Historically, heart injuries had fatal outcomes and were considered untreatable [3]; even today, about 90% of the patients die before reaching the hospital [4–6]. Different case series have reported survival rates ranging from 3 to 84% [6–9]. Some authors have found associations between mortality and patient’s hemodynamic status upon admission, kind of weapon used, wound characteristics, surgical findings, and complexity of the repair [10, 11].

Trauma characteristics may change according to social context, for example in blunt chest trauma, which is more frequent in developed countries, 30% of cardiac compromise has been reported. Survival rate for patients admitted to emergency departments in a shock state after PCI is about 35%, while for blunt chest trauma this rate is about 2% [3]. In the USA, the ratio between PCIs from gunshots (PCI-GSW) and from stabbing (PCI-SW) is 2:1 but in developing countries the latter is more frequent [1, 6]. These differences may influence the results of reported series.

Armed conflict and urban violence in Colombia generate a high incidence of traumatic injuries, but there are few reports in the literature about experiences in their management. The series from Hospital San Juan de Dios in Bogotá in the 1980s [12] and from Hospital San Vicente de Paul in Medellín in the 1990s are the two main studies of cardiac trauma in our country [13]. Our study reports 10 years of experience in managing PCI patients on a Level III institution and trauma referral center in Bogotá, with the objective to compare and analyze it against other series reported in literature and to describe particularities encountered on our experience.

## Methods

A cross-sectional retrospective case series of penetrating cardiac injury patients was done. Clinical charts of the patients with penetrating cardiac trauma that arrived to Hospital Occidente Kennedy (HOK) and underwent surgery from January 1999 to October 2009 were reviewed. Trauma team of HOK emergency service evaluated the patients, and upon admission, they were classified according to their hemodynamic status as proposed by Ivatury et al. [14]. Resuscitation was done according to the Advanced Trauma Life Support (ATLS) protocols of the ACS [15]. Patients dead upon arrival to the institution and those with Grade I cardiac wounds were excluded. Agonic patients and those with cardiac arrest were transferred immediately to an operating room for a resuscitative thoracotomy. Patients that arrived in deep shock (SBP ≤80 mmHg after reanimation with 2000 cc crystalloids) or with signs of cardiac tamponade [16] were submitted to a closed tube thoracostomy, median sternotomy, or thoracotomy (left or right), depending on the location of the wound and clinical and in some cases post-thoracostomy findings. In the initial years of the case series, the hospital did not have a permanent echocardiography service, and later on, the echocardiogram was introduced with academic purposes but was not used for the assessment of trauma patients in the emergency department protocols; therefore, none of the patients in this study received echocardiographic evaluation. Subxiphoid pericardial window was performed for all hemodynamically stable patients with injuries in the precordial region [9, 11] to rule out cardiac compromise. The Organ Injury Scaling of the American Association for the Surgery of Trauma (OIS-AAST) classification system [17] was used for cardiac injury grading (see Table 1). Repair method was selected according to hemodynamic status, associated lesions, and surgeon preference. The following additional data was obtained: age, sex, injury characteristics, injury-surgery time, and intensive care unit (ICU) and hospitalization time stay.

**Table 1:** Cardiac injury grading according to OIS-ASST system (see reference [17])

Grade	Injury description
I	Blunt cardiac injury with minor ECG abnormality (non-specific ST or T wave changes, premature atrial and ventricular contraction, or persistent sinus tachycardia). Blunt or penetrating pericardial wound without cardiac injury, cardiac tamponade or cardiac herniation.
II	Blunt cardiac injury with heart block (right or left bundle branch, left anterior fasicular or atrioventricular) or ischemic changes (ST depression or T wave inversion) without cardiac failure. Penetrating tangential myocardial wound up to but not extending through the endocardium, without tamponade.
III	Blunt cardiac injury with sustained (≥5 beats/min) or multifocal ventricular contractions. Blunt or penetrating cardiac injury with septal rupture, pulmonary or tricuspid valvular incompetence, papillary muscle dysfunction, or distal coronary arterial occlusion without cardiac failure. Blunt pericardial laceration with cardiac herniation. Blunt cardiac injury with cardiac failure. Penetrating tangential myocardial wound up to but not extending through the endocardium, with tamponade.
IV	Blunt or penetrating cardiac injury with septal rupture, pulmonary or tricuspid valvular incompetence, papillary muscle dysfunction, or distal coronary arterial occlusion producing cardiac failure. Blunt or penetrating cardiac injury with aortic or mitral valve incompetence Blunt or penetrating cardiac injury of the right ventricle, right atrium, or left atrium
V	Blunt or penetrating cardiac injury with proximal coronary arterial occlusion Blunt or penetrating left ventricular perforation Stellate injuries <50% tissue loss of the right ventricle, right atrium, or left atrium
VI	Blunt avulsion of the heart: penetrating wound producing >50% tissue loss of a chamber

Data from clinical records was collected manually using an instrument designed for that purpose, and then an EXCEL® database was created. The data was analyzed statistically using the IBM SPSS Desktop 20.0 for Windows. For quantitative variables, the mean, median, standard deviation, or range were used depending on the symmetry of data distribution. Qualitative variables, expressed in categories, were described as proportions, and to establish comparisons between proportions, the *z* test and Fisher exact test were used. A Type I error of less than 5% was accepted as being statistically significant.

## Results

Diagnosis of PCI was done in 308 cases according to the manual registration of the surgical unit. After reviewing the clinical charts, a total of 68 cases were excluded: 22 were Grade I cardiac injuries (exclusive of pericardial compromise), 13 cases had insufficient information in the medical records, and 33 had the clinical information which did not coincide with the PCI diagnosis. A final sample of 240 cases was reached.

The mean age was 27.8 years (SD = 9.1); most of the patients were males (*n* = 231; 96.2%). Overall mortality was 14.6% (*n* = 35). There was a total of 223 PCI-SW cases (93%) with a mortality of 11.7%. Among the 17 PCI-GSW cases, mortality was 41.2%. In 150 cases (62.3%), a pericardial window was performed for diagnosis (11 of the PCI-GSW and 139 of the PCI-SW). According to the hemodynamic classification from Ivatury et al. [14], 44% (*n* = 106) of the patients were on a normal hemodynamic status upon admission, 34% (*n* = 82) were in profound shock, 18% (*n* = 44) were in extremis or agonic, and 3% (*n* = 8) were dead on arrival. Signs of cardiac tamponade was found in 67% (*n* = 161) of the cases, and a similar distribution was found for both injury mechanisms (67% of the PCI-SW and 65% of the PCI-GSW cases). The mean time interval between the injury and surgical procedure was 60 min. In 73.6% of the cases, that time was less than 120 min. The median stay in the ICU was 5 days (range 1–30), and the median hospital stay was 6 days (range 1–58).

Based on the OIS-AAST system [17], 33% (*n* = 79) of the patients had Grade II injuries on arrival and a mortality of 2.5% (*n* = 2 of 79); 13.3% (*n* = 32) Grade III with a mortality of 12.5% (*n* = 4 of 32); 29.2% (*n* = 70) Grade IV and a mortality of 20% (*n* = 14 of 70); and 24.5% (*n* = 59) Grade V and mortality of 25.4% (*n* = 15 of 59). There was compromise of the right ventricle in 53% of the cases, the left ventricle in 32%, the right atrium in 10%; and the left atrium in 5%. There was simultaneous injury of two chambers in 4 cases (1.6%) and two or more injuries in one cavity in 12 patients (5%). Of the 106 patients admitted with a normal hemodynamic status, 71 (67%) had Grade II cardiac injuries; 15 (14%) had Grade IV; and 13 patients (12.5%) had Grade V. In 45% (*n* = 108) of the cases, there was only cardiac lesions.

### Type of weapon, surgical approach, and outcome

Mortality for PCI-SW group admitted with a normal hemodynamic status was 1% (1/99) whereas in the PCI-GSW group was 28.6% (2/7). The highest proportion of deaths occurred among individuals with Grade V injuries (12/53, 22.6%); whereas in the PCI-GSW group, 3 of the 4 patients with Grade IV wounds (75%) died (Table 2). The most frequently compromised cavities were the ventricles (*n* = 204, 85%), 127 (52.9%) cases in the right ventricle and 77 (32.1%) in the left.

**Table 2:** Distribution of patients according to gender, hemodynamic status on admission, wound classification, surgical intervention, and post-discharge conditions related to the mechanism

Wound type	Variables		Dead	Alive	Total
Stab wounds (*n* = 223)	Sex	Male	25	190	215
		Female	2	6	8
	Hemodynamic status	Fatal	2	6	8
		In extremis	8	32	40
		Deep shock	16	60	76
		Normal	1	98	99
	Wound classification	Grade II injury	0	73	73
		Grade III injury	4	27	31
		Grade IV injury	11	55	66
		Grade V injury	12	41	53
	Surgical approach	Sternotomy	9	135	144
		Thoracotomy—anterolateral	18	60	78
		Clamshell incision	–	1	1
		Thoracotomy—posterolateral	–	98	98
Gunshot wounds (*n* = 17)	Sex	Male	8	8	16
		Female	0	1	1
	Hemodynamic status	Fatal	0	0	0
		In extremis	4	0	4
		Deep shock	2	4	6
		Normal	2	5	7
	Wound classification	Grade II injury	2	4	6
		Grade III injury	0	1	1
		Grade IV injury	3	1	4
		Grade V injury	3	3	6
	Surgical approach	Sternotomy	3	5	8
		Thoracotomy—anterolateral	5	3	8
		Clamshell incision	NA	0	0
		Thoracotomy	NA	0	0

*NA* not applicable

### Mortality

**Fig. 1:**
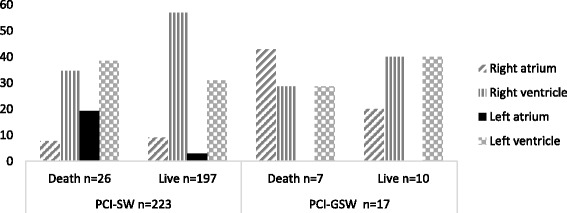
Distribution of the patients according to weapon type, localization of wound, and mortality

In right-ventricle injuries, mortality was 10.2% (13/127) and in left ventricle, 15.6% (12/77). Of the 18 injuries in the atriums, 7 occurred in the right atrium, of which 5 (71%) were fatal; of the 11 injuries in the left atrium, there were also 5 deaths (45.5%) (Fig. 1).

In 67.1% of the cases, there was cardiac tamponade. Mortality was 20.5% (33/161). Of the patients with tamponade, 93.2% had PCI-SW with 18% (27/150) mortality. Of the 11 PCI-GSW patients with tamponade, mortality was 54.5% (6/11). When comparing the proportions of individuals who died with tamponade injuries based on weapon type, there was a statistically significant difference (*p* = 0.0120). Of the 56 individuals remitted to the ICU, only 2 died.

## Discussion

In our series, young males predominate, similar to what has been reported in the literature. In contrast, trauma characteristics, treatment, and results had some differences. The more prevalent wound mechanism was PCI-SW (93%); at admission, a total of 106 patients (44%) had a normal hemodynamic status and 161 (67%) had cardiac tamponade. The high percentage of normal hemodynamic status patients partially explains the low overall mortality in this series (14.6%) compared to what has been reported in the literature [4–6, 18–24]; nonetheless, it is similar to the reports done by Villegas (10.4%) and Duque (13%) in Colombia [25, 26]. Mortality for the PCI-SW cases was 11.7% while in other series it ranges between 13 and 78% [2, 5, 10, 23–25]. For the PCI-GSW, mortality was 41.2%, similar to what is reported in series where type of injury predominate (26–85%) [2, 5, 10, 21, 23–25].

When analyzing the differences in mortality between wound mechanism, we could not identify significant differences in the prevalence for Grades II and III injury for PCI-SW and PCI-GSW cases (46.6 and 41.2% respectively); thus, we inferred that the higher mortality in PCI-GSW was due to not receiving early hospital care rather than injury grade. Another possible explanation for the high mortality is that most of PCI-GSW cases had associated lesions: thoracoabdominal (52.9%), chest (18%), and abdominal (1 case). This observation is consistent with other experiences like those from Asensio et al. [10], Buckman et al. [27], and others who have reported that associated lesions were a poor prognosis factor for PCI patients [28–30]. It is important to remark that in our center a deep analysis was not done to stablish the final cause of death (if heart wound or associated lesions) as this process in Bogotá must be done by specialized centers in forensic medicine.

The degree of hemodynamic compromise in this series also failed to explain the difference in mortality given that 78% of the PCI-SW and 76% of the PCI-GSW were admitted with a hemodynamic status ranging from normal to profound shock. This could be explained probably due to the high prehospital mortality rate in the latter group.

Although the proportion of patients with cardiac tamponade was similar for both types of injuries, it should be noted that when there was cardiac tamponade mortality was higher than the overall mortality (20.5 vs. 14.6%). Some authors consider cardiac tamponade to be a protective factor for patient’s survival [16]. Our results showed that the 2 of the 33 patients that died and had cardiac tamponade also had a normal hemodynamic status; moreover, 66% (*n* = 22 of 33) had some associated injury in the thorax or abdomen. It is important to point out that, in terms of physiopathology, filling of the pericardium space limits the stroke volume so in response cardiac frequency and right heart filling pressures are elevated through catecholamine production until right heart’s distensibility limit is reached, septum is pushed to the left side, and left side’s function is finally compromised [1]. For this reason, the longer the decompression of the pericardial space is delayed, a poorer prognosis should be expected which may explain our findings. As far as we know, the exact point when tamponade becomes a detrimental factor has not been stablished [27].

This series is unique in that a pericardial window was performed as the diagnostic procedure in 62.3% of the patients. As explained before, no echocardiographic assessment was done in this series, thus the protocol was to perform diagnostic pericardial windows in patients admitted with a PCI and a normal hemodynamic status. The group’s experience with this procedure was satisfactory, and the results were analyzed and published [31]. In none of our cases was the pericardial sac washed to define the management as some groups propose [32], which in hindsight could have prevented the thoracotomy done in the 22 cases (8.4% of the total series) excluded from the analysis for having only the pericardium compromised.

The frequent use of pericardial window partly explains our group’s preference for using sternotomy as the surgical approach and the good results obtained, similar to other series [29, 33]. In this series, a left anterolateral thoracotomy was generally used for resuscitation in patients with evident cardiac tamponade or in extremis. We concur that (i) the advantages of a sternotomy are better access to the right chambers and right pulmonary hilum, allowing cannulation for a cardiopulmonar bypass and (ii) that the left anterolateral thoracotomy facilitates access to posterior structures such as the esophagus, descending aorta, or left hilum [3]. Nevertheless, the choice of surgical approach also depends on the surgeon’s experience, the expected injuries according to the probable trajectory of the wounds, and the evidence of associated lesions.

Different physiological indexes and trauma mechanism have been proposed as predictors of mortality. Among the factors that affect survival rate after a PCI are the type of weapon used, the size of myocardial injury, cardiac injured chamber, compromise of coronary arteries, initial hemodynamic status, associated lesions, and the time elapsed until reaching the hospital, factors with which our series coincide. The overall most common wound location was the ventricles (*n* = 204), and compromise of the right and left ventricle was observed in 53 and 32% respectively, with a 10–15% mortality. In contrast, of the 18 injuries to the atria, 7 occurred in the right atrium with 71% mortality and 11 in the left atrium with 5 deaths (45.5%). It was surprising that some Grade IV and V patients were admitted with a normal hemodynamic status. Despite an overall survival rate of about 85%, it is not possible to address the “benevolence” of the cardiac injuries as this and other series are based on analyses of patients that received medical attention and thus represent the cases with better prognosis.

The average time between the moment of injury and execution of the surgical procedure was 60 min, and in 73.6% of the cases, this time was less than 120 min. It can be inferred that a large number of victims die before reaching the hospital. In a previous work by Pedraza and Isaza et al. (found in the Universidad del Rosario repository under the title Caracterización de la mortalidad por trauma cardíaco penetrante en Bogotá), 127 autopsies from individuals with heart injuries were performed finding that 71% died within the first hour and that only 51% received some type of medical attention.

The clinical follow-up was done until they were discharged from the hospital. The population’s socioeconomic conditions, the irregular availability of echocardiography, and the poor clinical ambulatory follow-up made it impossible for us to gather information on any residual intracardiac injuries; however, other studies have found a prevalence of valvular incompetence or septal ruptures in 19% of the cases [25, 29]. The hospital currently has stablished protocols for the clinical follow-up with electrocardiogram and strict imaging in PCI patients.

This study has limitations given that (i) it is a series of retrospective cases conducted in just one institution, where the majority of the patients come from a location with one of the highest indices of violence in Bogotá, which gives an atypical demographic profile; and (ii) the design does not permit a more in-depth statistical analysis. Nevertheless, the results permit us to generate new working hypotheses such as the need for revising the classification system of these injuries and the need of analyzing mortality from a forensic perspective.

## Conclusions

Despite the study limitations, we believe the number of cases included is of great interest in the cardiac injury experience and evidence. Several interesting findings were achieved in this series. High prehospital mortality rates in PCI patients was evidenced; the difference in mortality between PCI-SW and PCI-GSW was not associated with cardiac injury grade as should be expected; an unexpected higher mortality associated to cardiac tamponade, according to what has been described in literature of PCI, was found; and that pericardial window and sternotomy were a satisfactory diagnostic and surgical approach respectively. These findings suggest that adequate and early prehospital approach is essential for reducing mortality in PCI patients. Actual cardiac injury grading requires a further revision for improving accuracy in mortality prognosis for PCI patients. Additionally, a better comprehension of cardiac tamponade pathophysiology is needed to understand when and how a factor can be a protecting or a risk factor. We also believe more autopsy studies of PCI patients could help answering lots of these questions and improving the appropriate approach and management in the future.
